# Inhalation Administration of Agarwood Incense Rescues Scopolamine-Induced Learning and Memory Impairment in Mice

**DOI:** 10.3389/fphar.2021.821356

**Published:** 2021-12-24

**Authors:** Muxuan Han, Hao Zhang, Minghui Hu, Wei Sun, Zifa Li, Guimao Cao, Xiwen Geng, Sheng Wei

**Affiliations:** ^1^ College of Health Sciences, Shandong University of Traditional Chinese Medicine, Jinan, China; ^2^ Experimental Center, Shandong University of Traditional Chinese Medicine, Jinan, China; ^3^ Key Laboratory of Traditional Chinese Medicine Classical Theory, Ministry of Education, Shandong University of Traditional Chinese Medicine, Jinan, China; ^4^ TAIYUE Postdoctoral Innovation and Practice Base, Jinan, China; ^5^ Department of Anesthesiology, Affiliated Hospital of Shandong University of Traditional Chinese Medicine, Jinan, China

**Keywords:** agarwood incense, inhalation administration, learning and memory impairment, scopolamine, donepezil, dementia, intelligence-enhancement effect

## Abstract

**Background:** Agarwood, a type of herbal medicine widely used in Asian countries, is noted in traditional medicine for its intelligence-enhancing effects. Agarwood incense is traditionally administered by oral and nasal inhalation. To verify whether agarwood incense can exert its intelligence-enhancing effects in this way to rescue learning and memory impairment, typical clinical manifestations of dementia, we conducted a set of behavioral tests related to learning and memory.

**Methods:** C57BL/6 mice were divided into six groups. In addition to the control and model groups, we added a donepezil treatment group to evaluate the effect of three different agarwood administration doses. After a week of administration, scopolamine was injected 30 min before each behavioral test to create a learning and memory impairment model. A series of behavioral tests [the Morris water maze test (MWM), the novel object recognition test (NOR), and the step-down test (SDT)] were used to assess their learning ability, as well as their spatial and recognition memory.

**Results:** After scopolamine injection, the model group showed significant learning and memory impairment (i.e., longer latencies, lower crossing times, and lesser distance travelled in the target quadrant in MWM; a lower recognition index in NOR; and longer latencies and higher error times in SDT). The other four treatment groups all showed improvements in these indicators, and the overall therapeutic effect of agarwood was superior.

**Conclusion:** The inhalation administration of agarwood can significantly improve the learning and memory impairment caused by scopolamine in mice, and the therapeutic effect varied between doses.

## Introduction

Learning and memory impairment are major clinical manifestations of various brain diseases, including Alzheimer’s disease, and this greatly affects the quality of life of patients, causing a series of social problems (Jahn, 2013). Therefore, finding a drug that can treat it quickly, efficiently, and accurately is a pressing issue for the entire medical community. Currently, the commonly used western medicine preparations have limited efficacy and large side effects in clinical practice (Cummings et al., 2019), while complementary and alternative therapies are attracting increasing interest for their greater efficacy and fewer side effects when compared to western medicine (Shi et al., 2017).

Agarwood, a traditional sedative drug used in Asian areas, has a long history, and it is stated that it can cure “deficiencies of the heart and mind” in the Compendium of Materia Medica, which suggests that it may possess intelligence-enhancing properties. Modern pharmacological studies have shown that agarwood has a wide range of pharmacological effects such as anti-inflammatory, analgesic, and neuroprotective functions (Huo et al., 2017; Tan et al., 2019; Peng et al., 2020; Mi et al., 2021). The traditional method of using agarwood for therapeutic purposes is to heat it to cause it to emit volatile gases that are inhaled through the mouth and nose. Besides, as a sedative drug, the agarwood is also considered by traditional Chinese medicine to have aromatic resuscitation effects, which means that the agarwood is not only aromatic but also has an orifice opening effect. And if you use the theory of traditional Chinese medicine to explain the cause of learning and memory impairment, it is the filth in your body that clouds your heart and mind. The agarwood has the ability of dispelling the filth through its orifice opening effect, and oral and nasal inhalation administration can take advantage of the volatility of incense to maximize this effect. Based on these effects and on the classics of traditional medicine, we speculate that agarwood incense may have an intelligence-enhancing effect when administered by oral and nasal inhalation.

In this study, we used mice with learning and memory impairments induced by scopolamine (Flood and Cherkin, 1986), to verify whether agarwood incense, through oral and nasal inhalation, has efficacy for improving learning and memory as described in the classics of traditional medicine. We did this by using three behavioral tests—the Morris water maze test (MWM), the novel object recognition test (NOR), and the step-down test (SDT).

## Materials and Methods

### Animals

A total of 42 male C57BL/6 mice (18–22 g, 6 weeks old) were purchased from Vital River Laboratories (Beijing, China) [Laboratory animal production license number: SCXK (Jing) 2016-0006]. The animals were randomly divided into six groups: control group, model group, donepezil group, and agarwood high, medium, and low dose groups, and every three or four animals were housed in a cage with ad libitum food and water under controlled temperatures and 12-h light/dark cycles (lights on from 09:00 to 21:00 h) in a barrier environment [Laboratory animal use license number: SYXK (Lu) 2017-0022]. Prior to the experiment, the animals were adapted to the new laboratory environment for 7 days. The animal study was reviewed and approved by the Ethics Review Board of the Shandong University of Traditional Chinese Medicine [No. SDUTCM20210806007].

### Administration and Modeling Method

The administration of the agarwood tablets was conducted at 9:00 a.m. every day. The agarwood tablets (Chi-Nan fragrance, purchased from the Shandong Taiyue Biotechnology Co., Ltd.) were placed in the animal fragrance exposure system in the order of one, three, and nine tablets, and heated at 150°C for 5 min. This system has two units, one with a heating plate to heat the agarwood tablets to release the incense, and the other to hold the mice for administration. The two units are connected by rubber hoses and also have a gas exchange pump to discharge the exhaust gases. After the concentration was stable (concentrations of 1.5, 4.3, and 12.8 ppm), the mice in the low, medium, and high dose groups were placed separately in the system for oral and nasal inhalation for 20 min.

The other groups were subjected to the same conditions, except for the inhalation. In addition, the donepezil group was intraperitoneally injected with donepezil hydrochloride, which was dissolved in saline, at the same time (purchased from the Shanghai Macklin Co., Ltd.) at 3 mg kg^−1^ (Shin et al., 2018).

We choose scopolamine, an acetylcholine M receptor antagonist, to create a learning and memory impairment model. In addition to the control group injected with the same volume of saline, mice in the other groups were intraperitoneally injected with scopolamine (purchased from the J&K Scientific Co., Ltd.), which was dissolved in the saline too, at 3 mg kg^−1^ 30 min prior to each behavioral test [(Bertaina-Anglade et al., 2006; More et al., 2016)]. And mice were given 3 days for recovery and rest between each of the two behavioral tests, between which time they were no longer injected with scopolamine, but were administered as described previously.

### Behavioral Analysis

**FIGURE 1:**
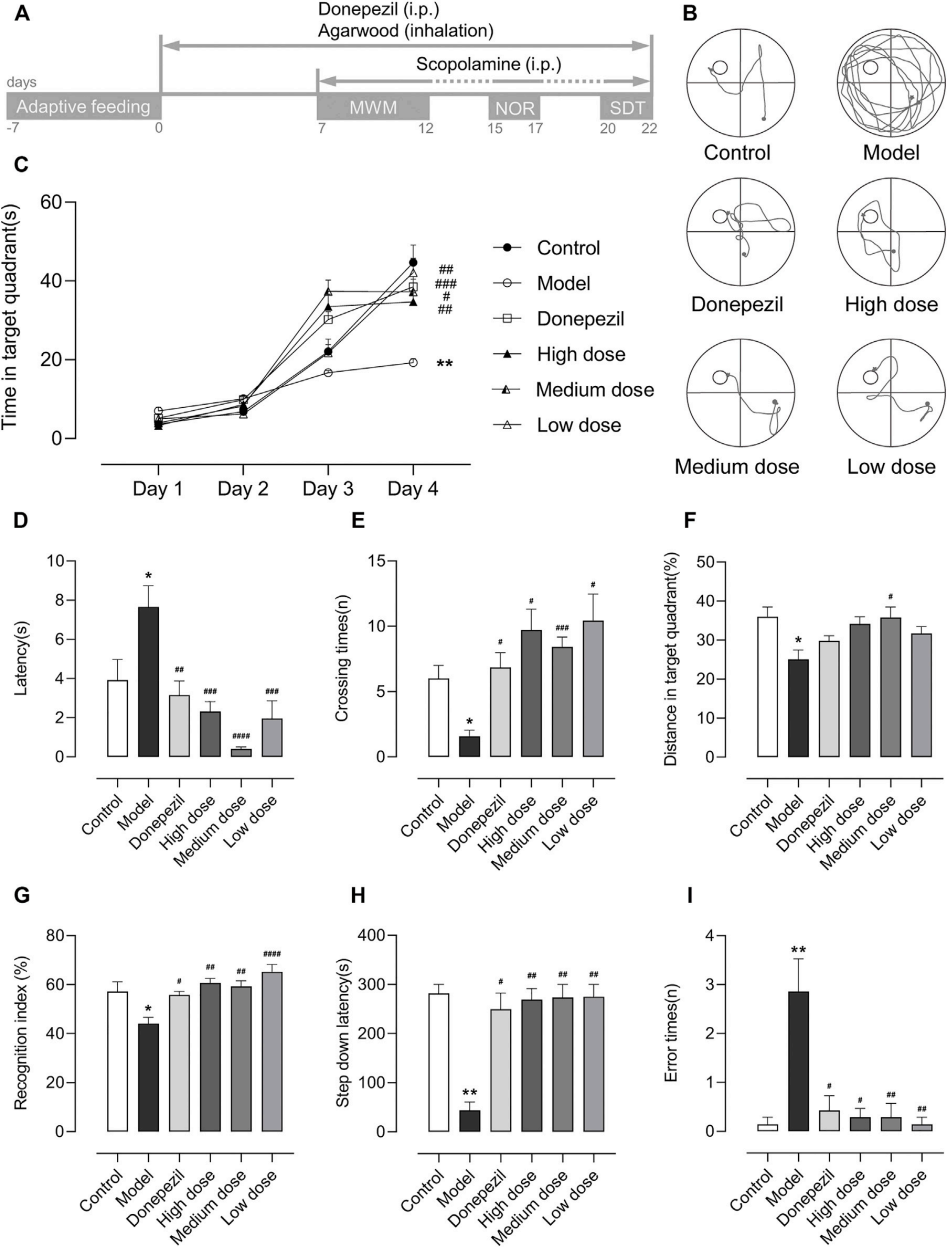
Schedule of treatment and behavioral tests and the results of the MWM, NOR, and SDT (*n* = 7). Data are shown as mean ± standard error of the mean (SEM). **(A)** After 7 days of adaptive feeding, the mice received a 22-days treatment except for the control and model groups, and then scopolamine was injected intraperitoneally 30 min before the start of each behavioral test except for the control group. Between each behavioral test, the mice were given 3 days to recover and did not need to be injected with scopolamine. **(B)** The trajectory on the fourth day of MWM. **(C)** The time in target quadrant for the first 4 days of MWM. (On the fourth day: Model: *p* = 0.008, Donepezil: *p* < 0.001, Agarwood high dose: *p* = 0.006, Medium dose: *p* = 0.023, Low dose: *p* = 0.004). **(D)** The escape latency of MWM. (F_5,36_ = 9.450. Model: *p* = 0.025, Donepezil: *p* = 0.004, Agarwood high dose: *p* < 0.001, Medium dose: *p*＜0.0001, Low dose: *p* < 0.001). **(E)** The crossing times of MWM. (F5,21.43 = 6.338. Model: *p* = 0.036, Donepezil: *p* = 0.028, Agarwood high dose: *p* = 0.019, Medium dose: *p* < 0.001, Low dose: *p* = 0.040). **(F)**. The distance in target quadrant of MWM. (F5,36 = 3.767. Model: *p* = 0.012, Donepezil: *p* = 0.635, Agarwood high dose: *p* = 0.05, Medium dose: *p* = 0.014, Low dose: *p* = 0.275). **(G)** The RI of NOR. (F5,36 = 7.242. Model: *p* = 0.016, Donepezil: *p* = 0.039, Agarwood high dose: *p* = 0.001, Medium dose: *p* = 0.003, Low dose: *p*＜0.0001). **(H)** The step-down latency of SDT. (Model: *p* = 0.001, Donepezil: *p* = 0.011, Agarwood high dose: *p* = 0.006, Medium dose: *p* = 0.002, Low dose: *p* = 0.002). (I). The error times of SDT. (Model: *p* = 0.003, Donepezil: *p* = 0.023, Agarwood high dose: *p* = 0.015, Medium dose: *p* = 0.004, Low dose: *p* = 0.003). (*p＜0.05, **p＜0.01 compared to the control group. #*p*＜0.05, ##*p*＜0.01, ###*p*＜0.001, ####*p*＜0.0001 compared to the model group).

Behavioral tests were performed from D7 to D22 (Figure 1A) between 13:00 and 18:00. And at 12:30, 30 min before the start of each behavioral test, including the training period of the tests, the mice were given an intraperitoneal injection of scopolamine.

In the MMW, we placed each mouse in a circular pool (80 cm in diameter) that was divided equally into four quadrants. The pool contained pigment that was the opposite of the mice fur color, thus making it easier for the camera on top of the pool to record the mice movements. For the first 4 days, a platform (8 cm in diameter) was placed in the middle of one of the quadrants with the top 2 cm below the water surface. The mice were then placed in the pool from each of the four quadrants in a random order. Each mouse had 2 min to find the platform, and if it was not found after that time, it was guided or placed on the platform for 20 s to form memories. On the last day, the platform was removed prior to the experiment, and the mouse was placed in the diagonal quadrant of where the platform was located for 2 min. We used the time in the target quadrant for the first 4 days as well as the escape latency, platform crossing times, and the relative distance in the target quadrant on the last day to evaluate their spatial memory storage and extraction ability. We also show the trajectory of the mice searching for the platform on the fourth day of the MWM (Figure 1B), and this allowed us to visualize the differences between the groups.

In the NOR, each mouse was placed in a chamber (50 × 50 cm) without any objects and allowed to explore freely for 3 min on the first day. After 24 h, the mice were placed with their backs facing two identical objects that were placed 10 cm away from the walls of the chamber and timed for 3 min. The number of times the mice explored each object was recorded. Then 1 h after completing this period, one of the two identical objects was replaced with a different one, and the mice were then placed as before for 5 min. Similarly, the number of times that the mice explored each object was recorded separately. We used the recognition index (RI), which is calculated as RI = the times of new object explored/(new object + old object) × 100%, to evaluate the recognition memory of the mice. As a rule, a higher RI represents a higher cognitive ability.

In the SDT, the testing apparatus consisted of five equal chambers (12 × 12 × 18 cm), each with an insulated platform (3× 3 × 3 cm) placed in the corner and an electrically energized copper grid (with 0.5 cm intervals) at the bottom. First, each mouse was placed in a chamber and allowed to habituate to the environment for 5 min, and then it was immediately electrified. The mice would jump on the platform to escape the electric shock. After 24 h, the mice were placed on the platform again for 5 min and electrified simultaneously. The time when the mice first jumped off the platform was taken as the step-down latency, and if it was greater than 5 min, it was recorded as 5 min. The number of times the mice jumped off the platform and suffered electric shocks was recorded as the error times. We used both indicators to examine the learning and memory abilities of mice under stressful stimuli.

### Statistical Analysis

Data analysis was conducted using GraphPad Prism 9 (GraphPad Software, La Jolla, CA, United States). For the time in the target quadrant in the MWM on the first 4 days, the comparisons between two groups were analyzed using a two-way analysis of variance (ANOVA) followed by Tukey’s test. Comparisons between the two groups of the other indicators in the MWM and NOR test were performed using a one-way ANOVA followed by Tukey’s test, except the data of the crossing times in the MWM, which was analyzed using the Brown–Forsythe and Welch ANOVA tests and Dunn’s test for its unequal standard deviation. The step-down latency and error times in the SDT were analyzed using the Kruskal–Wallis test followed by the Dunn’s test. *p*-values less than 0.05 were considered statistically significant.

## Results

### Intraperitoneally Injected Scopolamine Can Induce Learning and Memory Impairment in Mice

In all of the behavioral tests, the model group showed significant learning and memory loss compared to the control group after the scopolamine was injected (Figures 1C–I). They performed with longer latencies, had lower crossing times, and travelled lesser distance in the target quadrant (Figures 1D–F) in the MWM. This indicated that scopolamine caused damage to their spatial memory. Moreover, most of their trajectories indicated haphazard searches for the hidden platform (Figure 1B), and these types of search strategies did not improve significantly with more training as it did with the other groups (Figure 1C). In the NOR, as expected, the model group showed a lower RI (Figure 1G), indicating that the scopolamine also had a negative influence on the recognition memory of the mice. In addition, in the SDT, the longer latencies and higher error times (Figures 1H,I) of the model group also demonstrated emergency stimulus memory damage in the mice due to scopolamine. In addition, during the test, when the mice jumped down the platform and received electric shocks, we found an interesting occurrence as compared to the other groups. The mice in model group appeared to tolerate longer electric shocks. However, due to the limitations of the current instrumentation, we were unable to record and compare their reaction times accurately. However, we are keen to know whether this difference was related to scopolamine and, if so, whether it was due to the scopolamine-induced dementia that reduced their reaction speed.

### Inhalation Administration of Agarwood Incense Rescued the Scopolamine-Induced Learning and Memory Impairment in Mice

In the MWM, during the first 4 days as the training times increased, all of the groups exhibited varying degrees of prolonged time in the target quadrant (Figure 1C). In addition, during the last day of the test, all of the treatment groups showed significant improvements in the escape latencies and crossing times compared with the model group (Figures 1D,E). However, in the distance to the target quadrant (Figure 1F), the medium dose group showed a better treatment effect, while the other treatment groups showed a trend of increasing distances compared to the model group. The distance we used here was the ratio of distance in the target quadrant to the total distance travelled by the mice, as we found an increase in the total distance in the model group (Supplementary Figure S1A) along with a decrease in time in the target quadrant on the last day compared to the control group. Our explanation for this phenomenon is that the model group was unable to remember the location of the platform because of the spatial memory impairment caused by scopolamine. Hence, the mouse searched continuously throughout the pool, and this led to an increase in the total distance. However, because it searched each quadrant with similar frequency, it did not increase the distance travelled in the target quadrant (Supplementary Figure S1B). This also explains the difference in the time spent in the target quadrant on the last day, with the model group showing a significant decrease (Supplementary Figure S1C), while the high and medium dose of agarwood groups showed a significant increase in the time spent in the target quadrant.

In the NOR, compared to the model group, all of the treatment groups demonstrated a higher RI, which meant an improved cognitive performance in the mice (Figure 1G). In the SDT, all of the treatment groups showed both significantly longer latencies (Figure 1H) and lower error times (Figure 1I) compared to the model group. Here we found a possible quantitative dose–effect relationships relationship where the medium dose seemed to perform best in the MWM in all three agarwood groups, while the low dose seemed to show more therapeutic effects in the NOR and SDT, however, the high dose did not seem to perform more prominently in these behavioral tests. This suggests to us that it is possible that the best therapeutic dose exists between the low and medium doses, but because of the complexity of the agarwood incense composition and the different types of learned and memory ability that were assessed in the three behavioral tests, it is also possible that the medium dose is better for treating spatial recognition memory deficits than stimulus and object recognition memory, and vice versa for the low dose. However, based on the fact that both MWM and NOR are related to recognition memory, we prefer the former speculation, but of course further experiments are needed to verify the exact quantitative dose–effect relationship.

From the results it can be concluded that both agarwood and donepezil improved learning and memory abilities in the mice, but that the effect was more pronounced in the agarwood groups. These data suggested that through oral and nasal inhalation, agarwood incense rescued scopolamine-induced learning and memory impairment in mice.

## Discussion

In this study, the behavioral data we obtained suggested that through inhalation, agarwood incense had an intelligence-enhancing effect on learning and memory impairment in mice, as speculated.

Currently, research on agarwood is mostly focused on sleep disorders or anxiety-like behaviors [(Wang et al., 2017; Wang et al., 2018a)], and these are directly linked to traditional pharmacological effects, such as a sedative effect. However, only a few have improved learning and memory impairment. Sayyed Shahram Miraghaee (Miraghaee et al., 2011) studied the effect of agarwood incense smoke on the learning and memory ability of normal male Wistar rats, and showed that the agarwood did not improve spatial and visual recognition memories. But since they have selected only normal animals, instead of animals with learning and memory impairment to study on and used only the MWM to examine their spatial memory storage and extraction ability,is not that reliable. Except for the few studies on the effects of agarwood on learning and memory abilities, incense inhalation, a traditional and convenient method of drug administration, has received less attention than agarwood extracts like essential oil, which are typically administered by injection. Oral and nasal inhalation is more in line with the aromatic resuscitation effects of incense in Chinese medicine than injectable administration, and the results we obtained makes a good case for it.Our study fills the research gap in this area, confirms the intelligence-enhancing effects of agarwood incense, and provides a basis for further experimental studies and clinical applications

However, our experiment also has several flaws and shortcomings. First, there are many behavioral tests and mazes that can be used to test the learning and memory abilities of animals. However, due to time and equipment constraints, we chose a limited number of behavioral tests. In this experiment, only three of the classical tests, the MWM, the NOR, and the SDT, were performed to evaluate the spatial memory storage and extraction ability, cognitive memory, and contingency memory in mice. Second, there are many ways of administering agarwood, and many modern studies on its pharmacological effects have favored the injection of essential oils or extracts compared to the traditional oral and nasal methods of administration we used. We have not investigated whether different methods of administration affect the effect of agarwood incense on learning and memory impairment in mice. Finally, in this experiment, we used Chi-Nan fragrance agarwood tablets for administration, but studies have shown that the composition and content of different types of agarwood incense tablets are not identical (Yu et al., 2021). In addition, our experiment did not test incense tablets other than the Chi-Nan fragrance, so it is difficult to determine whether the effect of agarwood incense on learning and memory impairment in mice is related to its fragrance type.

In addition, there are still a few issues that we have not resolved in this experiment. Above all, the incense, which was released by heating agarwood and used for oral and nasal administration, was analyzed using gas chromatography-mass spectrometry (GC-MS) and liquid chromatography-mass spectrometry (LC-MS) techniques to confirm that the primary components were different types of 2-(2-phenylethyl) chromones and Flindersia type 2-(2-phenethyl) chromones [(Wang et al., 2018b; Peng et al., 2020; Yu et al., 2021)]. And the analyses result demonstrated that agarwood incense contains a variety of compounds, but it is still unclear which substance in the incense played a key role in this process. In our next experiments we will isolate the substances in the agarwood incense and investigate their effects on learning and memory impairment separately, and try to deduce which ones play the major role in rescuing learning and memory impairment in mice. This could also be the beginning of the development of a drug to treat diseases related to learning and memory disorders. Furthermore, according to the behavioral test data, we could see that the therapeutic effect did not correlate positively with the dose of agarwood incense as expected, and this was confusing. No obvious quantitative dose–effect relationships were seen, probably due to the complexity of the composition of the agarwood. Or perhaps the gradient intervals we set were too large to find the optimal doses, and we may need to adjust it to determine the optimal treatment dose. In addition, it is possible that there are different therapeutic effects of the different doses associated with the different types of learning and memory impairment, and we need to conduct further experiments that are targeted to focus on only one specific type of learning or memory disability to test this conjecture. Moreover, the mechanism by which agarwood incense improves scopolamine-induced learning memory impairment is still unclear. We have not yet conducted further studies to clarify the mechanism behind it. A possible cue is that the acetylcholine M receptors blocked by scopolamine play an important role in the central cholinergic neural mechanism that is related to learning and memory ((Hasselmo, 2006; Seifhosseini et al., 2011)). In particular, the M1 receptors. Because of the antagonistic effect of scopolamine on M receptors, we speculate that it is possible that agarwood incense alleviates this learning memory impairment by increasing the acetylcholine levels in the hippocampus and the cerebral cortex, while the M1 receptors was mainly distributed. And because of the sleep-enhancing effects of agarwood incense mentioned earlier, we hypothesize that the incense may also influence learning memory by modulating the levels of sleep-related monoamine neurotransmitters, such as 5-Hydroxytryptamine and Dopamine. Furthermore, as a traditional Chinese medicine, it is also possible that the agarwood may have a bi-directional effect depending on the dose. Based on our experimental results, it can be seen that the low and medium dose groups of agarwood treatment showed better improvement in learning memory deficits, while the high dose did not, which not only implies that there may be different regulatory mechanisms between different doses of agarwood but is also an indication of the importance of finding the optimal dose of agarwood for the treatment of learning memory disorders. These provide references for us to explore the possible relationship between agarwood incense and learning and memory, and to study the neural mechanisms behind them in the future.

## Data Availability

The raw data supporting the conclusion of this article will be made available by the authors, without undue reservation.
