# Supplementary material for: Inhalation Administration of Agarwood Incense Rescues Scopolamine-Induced Learning and Memory Impairment in Mice
Source: Front Pharmacol. 2021 Dec 24;12:821356. doi: 10.3389/fphar.2021.821356 (PMC8740194; doi:10.3389/fphar.2021.821356)
Supplement: Supplementary file 1 [file Image1.pdf]

Supplementary Material

1 Supplementary Figure 1

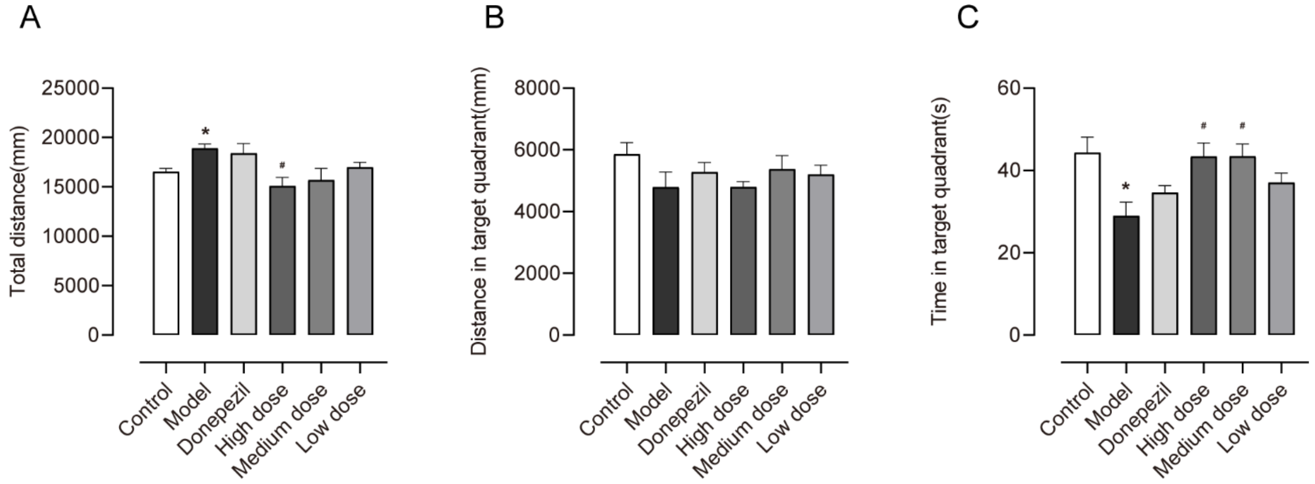

Supplementary Figure 1. Total distance travelled on the last day of MWM and distance travelled, and time spent within the target quadrant (n=7). Comparisons between two groups were performed using one-way ANOVA and followed by Tukey's test, except the total distance, which was using Brown-Forsythe and Welch ANOVA tests and Dunn's test for its unequal standard deviation. Data are shown as mean ± standard error of the mean (SEM). (A). The total distance. ($F_{5,22.97}$=3.609. Model: p=0.016, Donepezil: p>0.9999, Agarwood high dose: p=0.044, Medium dose: p=0.295, Low dose: p=0.149). (B). The distance in target quadrant. ($F_{5,36}$=1.197. Model: p=0.326, Donepezil: p=0.930, Agarwood high dose: p>0.9999, Medium dose: p=0.864, Low dose: p=0.965). (C). The time in target quadrant. ($F_{5,36}$=4.223. Model: p=0.011, Donepezil: p=0.770, Agarwood high dose: p=0.020, Medium dose: p=0.019, Low dose: p=0.421). (*p＜0.05 compared to the control group. #p ＜0.05 compared to the model group).
